# Maternal childhood maltreatment trauma resolution: Development of a novel narrative coding measure and implications for intergenerational parenting processes

**DOI:** 10.1017/S0954579423001256

**Published:** 2023-10-04

**Authors:** Hannah G. Swerbenski, Melissa L. Sturge-Apple, Grace Messina, Sheree L. Toth, Fred Rogosch, Dante Cicchetti

**Affiliations:** 1 University of Rochester, Rochester, NY, USA; 2 Mt. Hope Family Center, University of Rochester, Rochester, NY, USA; 3 University of Maryland, College Park, MD, USA; 4 Institute of Child Development, University of Minnesota, Minneapolis, MN, USA

**Keywords:** intergenerational transmission, maltreatment, parenting, trauma resolution

## Abstract

Child maltreatment constitutes a significant environmental risk for children, with carryover effects into future generations. There is a need to characterize protective factors that may buffer against the intergenerational transmission of maltreatment. The current study addresses this gap through two primary aims: 1) the development and validation of a novel measure assessing resolution of maternal childhood maltreatment trauma using narrative coding methods and 2) the evaluation of maternal maltreatment trauma resolution as a buffering factor that may moderate associations between maternal neglect histories and sensitive parenting of offspring. Results of reliability analyses from this sample of 210 diverse, low-income mothers suggest the novel childhood maltreatment trauma resolution measure is highly reliable. Furthermore, results highlight the generalizability, criterion validity, and concurrent and predictive validity of the measure. Results from cross-sectional analyses show that trauma resolution moderates associations between maternal physical neglect histories and sensitive parenting, such that under high maternal trauma resolution, there is no longer a negative association between neglect histories and sensitive parenting. Results from longitudinal analyses also show a protective effect of maternal trauma resolution, such that trauma resolution has a protective-enhancing effect on maternal sensitivity. Implications for research and clinical practice with families are discussed.

## Introduction

Child maltreatment constitutes a grave public health concern and particularly harrowing environmental risk for children. Millions of children are abused and neglected each year, impacting 1 in 4 children ages 0–17 (Afifi & Macmillan, 2011; Finkelhor et al., 2015). A wealth of previous research has established links between child maltreatment and deleterious outcomes throughout the lifespan, encompassing a range of psychological and physical health consequences (Carr et al., 2020; Cicchetti, 2016; Widom, 2014). Unfortunately, the negative effects of child maltreatment may also carry over into the next generation (Berlin et al., 2011; Greene et al., 2020). For example, mothers with childhood maltreatment histories have been found to exhibit less sensitive parenting than mothers without maltreatment histories (Pereira et al., 2012). Infants of maltreated mothers also have higher rates of insecure attachment relative to rates observed in the general population (Berthelot et al., 2015). Moreover, meta-analytic findings suggest continuity of maltreatment across generations (Assink et al., 2018; Madigan et al., 2019).

However, not all parents who were maltreated themselves go on to maltreat their own children, which underscores the importance of explicating factors that may reduce the intergenerational continuity of maltreatment (Cicchetti & Doyle, 2016; Langevin et al., 2021). Meta-analytic work examining associations between maternal childhood maltreatment histories and parenting reveals a small effect size in the association between childhood maltreatment and maternal parenting behavior (*r* = −.13; Savage et al., 2019). This suggests that there may be individual differences in the association between maltreatment histories and current parenting, whereby some mothers may be less vulnerable to maladaptive parenting outcomes following maltreatment. In order to identify individual differences that may explain discontinuity and foster intergenerational resilience, the present study examines how the ability to resolve trauma associated with maltreatment experiences may buffer against the spillover of negative sequalae into future generations.

### Maltreatment histories and parenting the next generation

Determinants of parenting process models provide a framework for understanding how maternal experiences and histories of caregiving may inform our understanding of the intergenerational transmission of maltreatment (Belsky & Jaffee, 2015; Belsky, 1984). Within these conceptualizations, parents’ ontogenic origins and personal psychological resources are thought to play an influential role in their current caregiving. Elaborating on the role of childrearing history, Belsky integrates tenets from attachment theory to suggest that maladaptive parenting is transmitted across generations via internal working models of the caregiving relationship. Internal working models are understood as dynamic representational systems that describe individual orientations towards and understandings of habitual social interactions (Bretheron & Munholland, 2016). In accord with this perspective, prior research (Korja et al., 2010) on maternal internal representations of infant offspring suggests that maladaptive maternal internal representations are linked to deficits in observed caregiving, including less positive communication and lower dyadic mutuality. This perspective dovetails with psychodynamic frameworks, which propose that adults’ representations of their caregivers are likely to increase their propensity to enact the same maladaptive parenting behaviors internalized from their own childhood (Iyengar et al., 2019). The ability to resolve trauma associated with one’s own negative and abusive caregiving experiences may serve as one avenue for modifying internal representations, and in turn, support positive caregiving in future generations. To date, researchers have not explicitly examined the degree of maternal childhood maltreatment trauma resolution in the context of maltreatment and the intergenerational transmission of parenting.

Innovative empirical work on acceptance in survivors of childhood sexual abuse suggests that resolution and acceptance may be an important process underlying resilient functioning following child maltreatment experiences (Noll et al., 2003; Noll, 2004). According to these accounts, healing from the trauma of childhood maltreatment may involve forgiveness and acceptance. For instance, Noll (2003) examined four aspects of forgiveness and acceptance (letting go of anger, cessation of revenge, conciliation, and moving on in life despite the offense) in a longitudinal study of sexual abuse survivors, finding that higher forgiveness was associated with higher self-esteem, and lower anxiety, depression, and posttraumatic stress disorder symptoms. These aspects of forgiveness were used in conjunction with psychodynamic and attachment-informed theories of parenting to design a novel, continuous measure of maternal childhood maltreatment trauma resolution. In the current study, *resolution of childhood maltreatment trauma is defined as the degree of acceptance and forgiveness presently held by the participant regarding the experiences and circumstances of their childhood upbringing*. Given that parental psychopathology is associated with difficulties in caregiving (Erickson et al., 2019), we hypothesize that the reduced psychopathology associated with higher levels of forgiveness and acceptance identified by previous research (e.g., Noll, 2003) may also confer benefits for caregiving.

In further support of these proposed caregiving benefits, we argue resolution of trauma associated with negative and abusive caregiving experiences may develop alongside *earned security*, which has been empirically linked to positive parenting outcomes (Saunders et al., 2011). The concept of earned security builds on work pioneered by Main and colleagues (e.g., Main et al., 1985; Main et al., 1984-2003) using the Adult Attachment Interview (AAI), a structured, semi-clinical interview focusing on early attachment experiences and their effects. This system assesses *coherence* of attachment states of mind, which refers to the ability to provide an elaborated, relevant, and consistent account of one’s relational caregiving experiences. Notably, it is possible to coherently reflect on both very positive and very negative caregiving experiences. Adults who coherently reflect on difficult childhood attachment experiences are understood as having achieved *earned security*. In turn, individuals with earned security have been found to demonstrate better parenting outcomes than insecure individuals, that is, those individuals who are unable to provide coherent accounts of their caregiving experiences (Saunders et al., 2011).

Relevant to the current investigation, we posit that in order to resolve the trauma associated with abusive caregiving experiences, one must be able to provide a coherent account of these negative caregiving experiences. Thus, earned security can be thought of as a necessary (but not alone sufficient) condition for earning high scores on the dimensional measure of resolution used in the current study. Beyond coherent reflection on caregiving experiences, the highest scores on the resolution code are reserved for mothers who demonstrate an understanding of how these negative childhood experiences impact their current feelings towards and relationship with their own caregivers, with the highest scores indicating high degrees of acceptance and even forgiveness towards their own caregiver (see Supplemental Materials for coding manual and sample excerpts). Similar to proposed theoretical mechanisms underlying earned security, we propose the process of grappling with these negative and abusive childhood experiences may confer benefits for caregiving through modifying internal attachment representations.

Finally, we review how the conceptualization and operationalization of resolution advanced in the current study address limitations of existing narrative measures of intergenerational attachment processes. Regarding measures of earned security, previous research (Pearson et al., 1994; Roisman et al., 2002) has found that “earned secures” report elevated depression symptoms relative to individuals who report more positive caregiving experiences growing up (e.g., “continuous secures”). Thus, it remains possible that the negative but coherent early caregiving experiences reported by earned secures on the AAI may actually reflect depression-related biases towards selectively remembering negative caregiving experiences during childhood (Phelps et al., 1998; Roisman et al., 2014). The limitations associated with the retrospective assessment of childhood attachment quality in earned security conceptualizations are further underscored by research from Roisman et al. (2002), which examined attachment experiences both prospectively and retrospectively using a 23-year longitudinal dataset. More specifically, Roisman and colleagues found that despite retrospectively reporting negative childhood experiences on the AAI, observational data collected during childhood actually indicated that “earned secures” had similarly positive caregiving experiences to continuous secures. By emphasizing levels of acceptance and forgiveness in the *current* relationship between the adult child and her caregiver, we cautiously suggest the coding system in the current study may at least partially offset concerns about the limitations of retrospective recall. However, given that current feelings of acceptance and forgiveness may concern events and transgressions from the distant past, limitations associated with retrospective recall should not be fully disregarded. Perhaps the most significant limitation of earned security conceptualizations involves the strict classification of individuals as insecures, earned secures, and continuous secures, which may artificially dichotomize a dimensional construct (see Roisman et al., 2002 for a more detailed critique). Likewise, limitations related to artificial dichotomization may also apply to other assessments, such as the coding of “unresolved” classifications on the AAI, which assesses lapses in the monitoring of reasoning or discourse in narratives around loss and abuse experiences. Additionally, both “unresolved” AAI classifications (e.g., Jacobvitz & Reisz, 2019; Madigan et al., 2012) and the Hostile/Helpless States of Mind Coding System (Lyons-Ruth et al., 2005; Terry et al., 2021) tend to emphasize deficits in functioning that may potentiate intergenerational transmission of maltreatment rather than focusing on fostering intergenerational resilience. This suggests a need for narrative measures that capture aspects of resilience promoting factors, such as resolution of maltreatment trauma. Thus, in contrast to existing measures, the childhood maltreatment trauma resolution measure used in the current study 1) assesses current, implicit processes to yield dimensional rating scores; 2) emphasizes resilience processes; and 3) integrates multiple perspectives that complement attachment theory, including determinants of parenting models and previous psychodynamic research on forgiveness.

### Applying resolution to an understudied context: Neglect histories and parenting

Beyond measure development aims, we also sought to empirically evaluate the potential protective effects of resolution for caregiving. In the applied portion of our analyses (Aim 2), we have intentionally chosen to focus on neglect and its intergenerational sequelae for three primary reasons: prevalence, lack of existing research on neglect, and developmental timing. Neglect refers to a failure to provide for a child’s needs (Mennen et al., 2010). First, data from CPS investigations suggest that neglect is the most common form of child maltreatment (Kim et al., 2017; Stoltenborgh et al., 2013). Families are also more likely to be repeatedly reported for neglect compared to other maltreatment subtypes (Jonson-Reid et al., 2019). Despite its prevalence, neglect is understudied relative to other maltreatment subtypes (McSherry, 2007; Mulder et al., 2018; Stoltenborgh et al., 2013). Regarding developmental timing, neglect is most prevalent during infancy (CDC, 2008). Increased rates of neglect during infancy may be related to the stress of the transition to parenthood. Further, research has identified associations between neglect histories and known risk factors for maladaptive parenting, including psychopathology and delinquency (Ryan et al., 2013; Warmingham et al., 2019), insecure attachment (Venet et al., 2007), substance abuse (Dunn et al., 2002), and impaired processing of facial expressions (Doretto & Scivoletto, 2018). Finally, infancy also represents a unique opportunity for identity reconstruction for parents (Christie et al., 2017; Raphael-Leff, 2001), suggesting infancy is also an important period for evaluating potential protective processes, such as trauma resolution.

**Figure 1.**
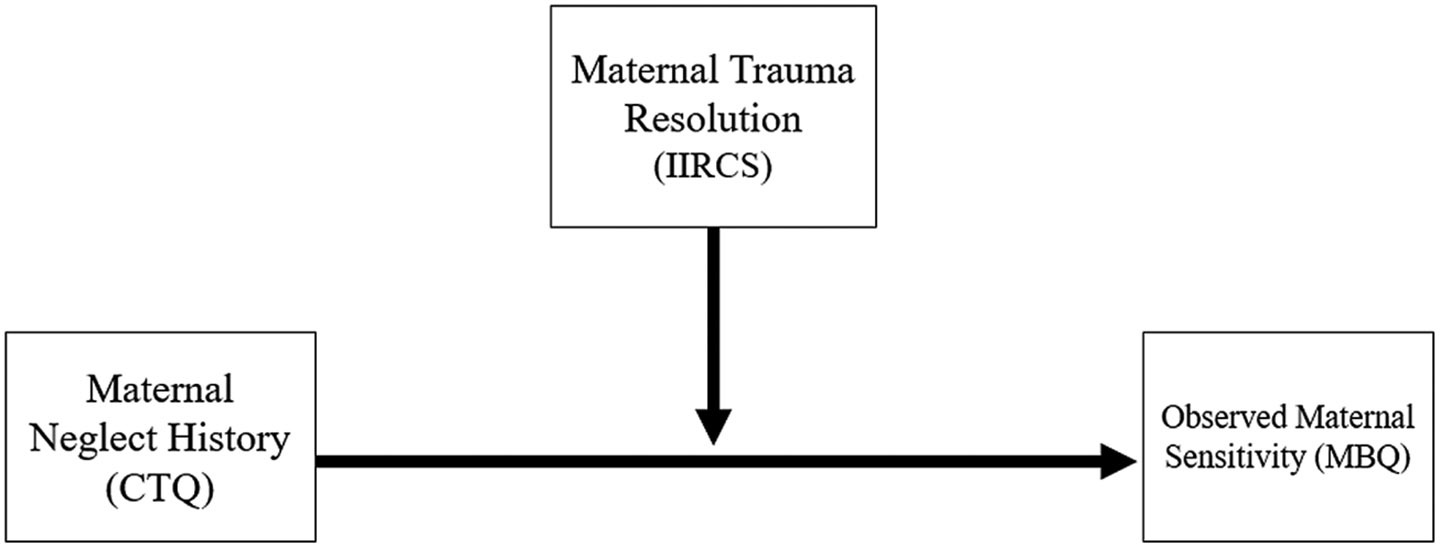
Conceptual model for Aim 2, which evaluates maternal trauma resolution as a buffering factor that may moderate associations between maternal neglect histories and observed parenting sensitivity.

In summary, the present study draws on Determinants of Parenting models and psychodynamic perspectives to propose a novel narrative measure of maternal maltreatment trauma resolution and evaluates the reliability and validity of this narrative coding approach (Aim 1). We report on interrater reliability, generalizability, and several forms of validity including criterion validity, convergent validity, concurrent validity, and predictive validity. This study also examined the buffering role of maternal maltreatment trauma resolution on the association between maternal neglect histories and sensitive parenting of offspring (Aim 2, See Figure 1).

## Methods

### Participants & procedures

Participants in the current study include 210 mothers and their 13-month-old infants (52.9% female). To qualify, infants were required to be living with their participating mother at the time of enrollment. This sample is diverse, with 53.8% of mothers identifying as Black or African American, 25.5% as White, 9.6% as Hispanic, 2.9% as Asian, and 4.8% as “other.” Maternal age ranged from 18 to 41 years (*M* = 26.9 years). Participants came from low-income backgrounds, with a mean total income of $10,330 when including public assistance. Regarding education, 41.8% of mothers reported that they did not complete high school, 38.5% had a high school degree or GED equivalent, 18.8% had at least some college experience, and 1% had a bachelor’s degree.

**Figure 2.**
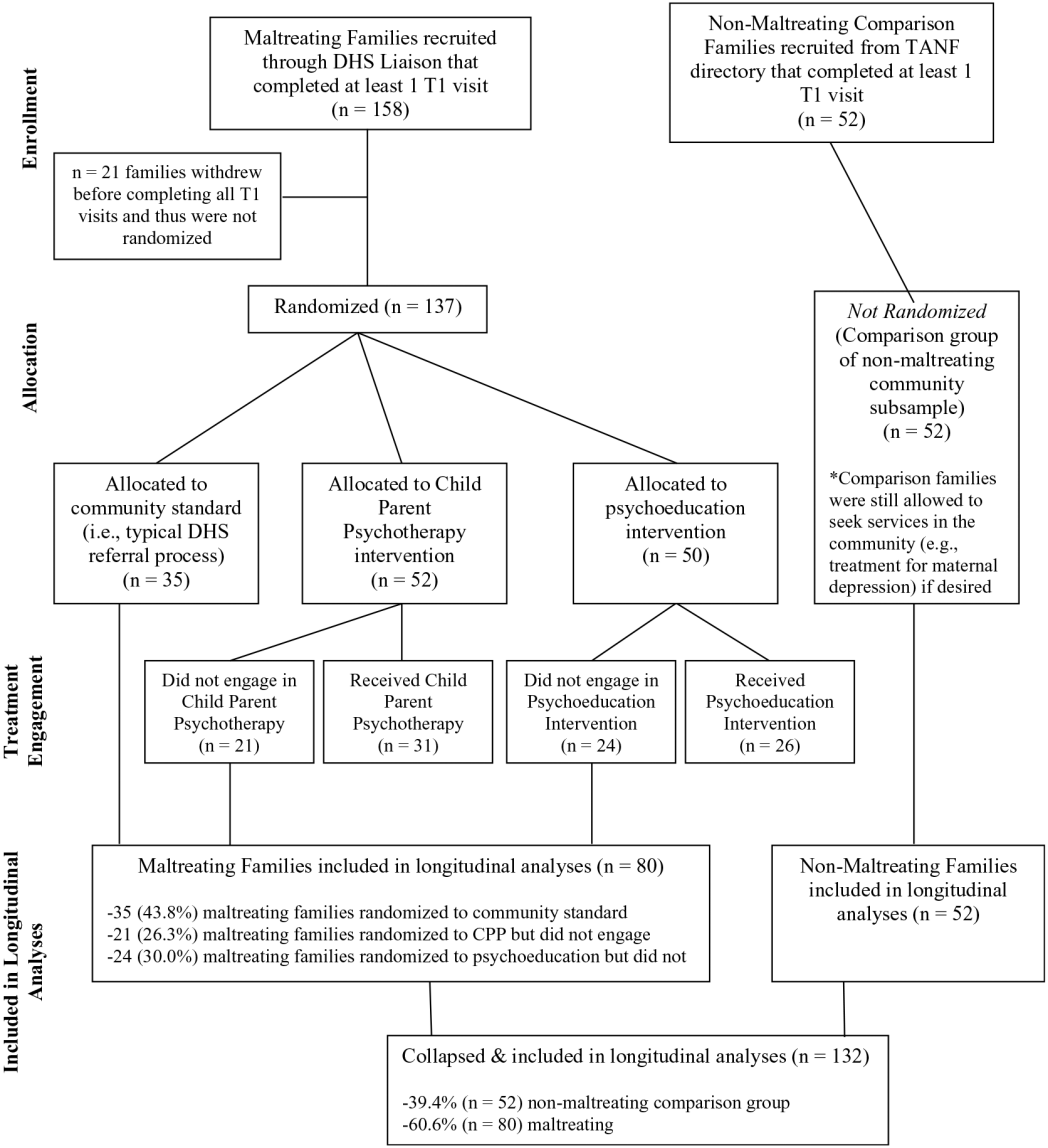
Flow chart diagram summarizing treatment groups and randomization for larger RCT design. DHS = department of human services. TANF = temporary assistance for needy families. CPP = child parent psychotherapy. Randomization occurred after the conclusion of all T1 visits to avoid any confounding effects associated with anticipated treatment. For the purposes of the current study, the collapsed group included in longitudinal analyses (*n* = 132) also includes families who never engaged in the treatment, as these families were not expected to show confounding treatment effects despite being randomized to treatment.

Participants were recruited as part of a larger randomized-controlled trial of a Child-Parent Psychotherapy intervention for high-risk mothers and neglectful families. As part of this larger study design, a majority (72.2%) of participating families were maltreating families from low socioeconomic status backgrounds, as substantiated in records with Child Protective Services (CPS). Of those families with CPS cases, all investigations had been closed by the time of their enrollment to avoid coercion, with relevant indicated reports and determinations complete. Inclusion criteria for the maltreated groups required that maltreatment occurred with the infant and/or the infant was living in a maltreating family with their biological mother. In the recruited sample, 66.4% of the maltreated infants were indicated as the targets of abuse and/or neglect, and 33.6% of the cases identified a sibling as the target. Recruitment of participants was facilitated by a Department of Human Services (DHS) employee liaison, who followed a recruitment script that assured families of confidentiality. The DHS liaison did not share any information with the project staff unless the family signed a release of information. Non-maltreating comparison families were recruited using a local directory of families receiving Temporary Assistance for Needy Families (TANF). Non-maltreating comparison families were socio-demographically similar to the maltreating families. Although the present analyses do not concern treatment effects, a diagram summarizing the breakdown of treatment and comparison groups in the larger RCT design is shown in Figure 2.

The current study utilizes data from T1 (Baseline) when infants were 13 months old and T3 when infants were 26 months old. Cross-sectional (T1) analyses include the full sample’s (*n* = 210) available data unless otherwise stated. In order to examine associations independent of intervention effects associated with the larger study design, longitudinal analyses included only participants who did not engage in treatment (*n* = 132, includes *n* = 87 in non-treatment groups and *n* = 45 who were randomized to treatment but never engaged). At both T1 and T3, mothers and infants completed five total visits (two home visits and three laboratory visits). The last home visit at both time points consisted of extended naturalistic observation by trained experimenters. All experimenters were trained in conducting sensitive interactions with families and mandated CPS reporting by on-site psychologists. Experimenters were also required to complete practice pilot visits with volunteers before interacting with participating families. All study procedures were approved by the Institutional Review Board of the University of Rochester and written consent was obtained from all participating mothers prior to study enrollment.

### Measures

#### Focal measure: Maternal childhood maltreatment trauma resolution

##### *Infant and intergenerational relationships coding system* (*IIRCS;* Swerbenski & Sturge-Apple, 2020).

This novel narrative coding scheme for the *Parent Development Interview* (*PDI*; Aber et al., 1985) assessed maternal relationships with her infant and her own caregivers. All participating mothers completed the PDI at T1 (Baseline) when infants were approximately 13 months old. The PDI is a validated, semi-structured interview which indexes parents’ mental states and representations of their relationship with their child and the parenting role. The PDI also features questions about the intergenerational relationship between the mother and her own parents and how her experiences during her upbringing affect her as a parent (e.g., “How do you want to be like and unlike your mother as a parent?”). The IIRCS includes 14 total codes which assess the mother-infant relationship and the mother’s relationship with her own parents respectively. The present analyses use only the resolution code, which assesses “the degree of acceptance and forgiveness presently held by the mother [towards her caregiver] regarding the experiences and circumstances of her childhood upbringing.” Coders rate each interview transcript using a dimensional scale from 1 (Not at all characteristic) to 9 (Mainly characteristic). Sample narrative excerpts indicative of a range of scores on this measure can be found in the supplemental materials. Maternal childhood maltreatment trauma resolution was coded for participants’ relationship with their primary female caregiver (94.8% of participants discussed their biological mother in the PDI, 4.3% discussed their grandmother, and the remaining participants discussed an adoptive mother or aunt). All interviews were transcribed verbatim and coded according to the IIRCS manual.

Coders received several weeks of training and were consistently monitored by the first author for coder drift and consistency. Coder training included theoretical and empirical readings relevant to the constructs coded, general background in narrative coding, and discussions of cultural sensitivity, particularly with respect to racial disparities in child maltreatment and child welfare involvement. Moreover, transcripts were de-identified and coders could not readily identify the race or ethnicity of participants. The three coders identified themselves as follows: 1) non-Hispanic White woman; 2) non-Hispanic multiracial (Black and White) woman; and 3) non-Hispanic White woman. These three coders rated a randomly selected subset of interviews (*n* = 44, 21.4%) for reliability purposes. Resulting reliability was excellent (*ICC* = .937). Additional indices of reliability, generalizability, and validity of this measure are presented in the results.

#### Validity measures

##### *Perceptions of adult attachment scale* (*PAAS;* Lichtenstein & Cassidy, 1991).

Participating mothers completed this 60-item self-report measure which assesses adult perceptions of early caregiving experiences and their current state of mind towards their caregiver at T1. Mothers rated each item on a 5-point Likert scale from “strongly disagree” to “strongly agree.” This measure has been shown to have good internal consistency (Lichtenstein & Cassidy, 1991).

The present study uses the *Balancing-Forgiving* subscale, which describes current states of mind towards one’s own caregiver, including forgiveness and current positive regard. Given that the *Balancing-Forgiving* scale is the best available existing self-report measure of acceptance and forgiveness towards caregivers, this subscale was used to evaluate the convergent, concurrent, and predictive validity of the novel resolution measure proposed in this study. Items for each of these subscales are included in the supplemental materials. The Cronbach’s alpha for the Balancing-Forgiving subscale of the PAAS suggests somewhat low internal consistency in the present sample (*α* = .593), although other subscales of the PAAS had higher internal consistency in this sample (αs = .731–.895).

##### *Parenting stress index (PSI;* Abidin, 1990).

The PSI is a 101-item questionnaire assessing parenting stress in the child domain (adaptability, acceptability, demandingness, mood, dis-tractibility, hyperactivity, reinforcing parent) and the parent domain (depression, attachment, restrictions of role, sense of competence, social isolation, relationship with spouse, parent health). The present study utilizes T1 scores on the *Total Stress* and the *Parenting Sense of Competence* subscales to assess concurrent validity. Internal consistencies for these subscales in this sample were excellent (Total Stress *α* = .95, Parenting Sense of Competence *α* = .90).

##### *Symptom checklist 90 revised* (*SCL-90-R*; Derogatis et al., 1973; Derogatis & Savitz, 1999).

Mothers completed the 90-item SCL-90-R at T3 when infants were approximately 26 months old. The SCL-90-R is a widely used, self-report symptom inventory that assesses patterns of current psychological symptoms. Each item is rated on a 5-point Likert scale from “not at all” to “extremely.” The current study used the *depression* and *anxiety T*-scores on the SCL-90-R to assess predictive validity of the resolution code. Normative data were used to construct *T*-scores based on participant gender and population (e.g., community sample). Normative *T*-scores have a mean of 50 (*SD* = 10). Mean scores in this sample were 57.91 and 51.89 for depression and anxiety respectively, which is in line with expectations for a higher risk community sample.

#### Maternal sensitivity

##### *Maternal behavior Q-Set* (*MBQ;* Pederson & Moran, 1995).

At T1 and T3, following a 3-hour home observation of mother–child interaction, two trained observers independently completed the MBQ. The MBQ consists of 90 items that assess features of maternal sensitivity. Observers sort the 90 items into a forced distribution of nine piles according to the extent to which the item is characteristic or uncharacteristic of the mother’s behavior. The distribution of items for each mother is correlated with an ideal criterion distribution of maternal sensitivity to derive an individual score. Thus, possible scores range from −1 to 1, where 1 indicates perfect correlation with the ideal criterion distribution. Intraclass correlations between pairs of observers were acceptable (*ICC* = .71). Maternal sensitivity was used both in concurrent validity analyses (T1) and in moderation analyses (T1 and T3).

#### Maternal neglect history

##### *Childhood trauma questionnaire* (*CTQ;* Bernstein et al., 1994).

Mothers reported on their own childhood maltreatment history using the CTQ, a 61-item self-report measure that indexes retrospective accounts of childhood maltreatment. The CTQ assesses five domains of maltreatment histories: emotional abuse, physical abuse, sexual abuse, emotional neglect, and physical neglect. The reliability and validity of these subscales has been affirmed by previous work (e.g., Bernstein et al., 2003). For the purposes of this study, we focused on *physical neglect*, which was indexed using a continuous, subscale score of items on the physical neglect subscale. Cronbach’s alpha for the physical neglect subscale of the CTQ for this particular sample was very good (*α* = .858).

#### Covariates & demographics

##### Sociodemographic covariates.

All mothers completed a brief demographics interview at T1, including items concerning race and ethnicity, socioeconomic status, marital status, and maternal age. Associations between demographics and resolution scores are presented in the first section of results (Aim 1). Concurrent and predictive validity analyses control for maternal age and education. Moderation models (Aim 2) control for maternal age.

##### *Wechsler adult intelligence scale* (*WAIS-III*; Wechsler, 1997).

All participating mothers completed the Vocabulary and Comprehension subtests of the WAIS-III at T1. A composite verbal ability variable was created from the average of these subtest scores. We control for verbal ability in analyses, as verbal ability has been shown to predict individual differences in scores on other narrative measures such as the Adult Attachment Interview (Crowell et al., 1996).

##### *Maltreatment classification system* (*MCS*; Barnett et al., 1993).

As part of the design of the larger study, mothers were classified as maltreating or non-maltreating at Baseline (T1). Maternal maltreatment perpetration was coded from records with the Department of Human Services using the MCS. The reliability and validity of the MCS has been demonstrated in previous research (Bolger et al., 1998; Manly, 2005). Based on operational criteria, the MCS identifies and designates various subtypes of maltreatment, including neglect, emotional maltreatment, physical abuse, and sexual abuse. Coding of DHS records was completed by trained research staff and clinical psychologists, demonstrating good reliability (weighted *k* = .86–.98). Of infants with documented maltreatment at baseline, 8.8% had been physically abused, 84.6% had been neglected, and 69.2% had been emotionally maltreated. None had been sexually abused.

## Results

### Data preparation

Prior to conducting focal analyses, all data were screened for outliers and continuous endogenous variables were screened for non-normality. No outliers were removed based on screening. No key variables exhibited significant skew nor did any variables exhibit significant kurtosis, with the exception of T3 maternal sensitivity, which had a kurtosis value of −1.13, indicating a slightly platykurtic distribution. However, given that 1) kurtosis was not observed in T1 sensitivity; 2) the kurtosis for T3 maternal sensitivity was mild; and 3) the Q-set used to assess sensitivity bases sensitivity scores on their correlation with an established criterion distribution that enhances interpretation, we opted not to transform the maternal sensitivity data.

All reliability and validity analyses were conducted in IBM SPSS Statistics 26. These include analyses to evaluate reliability and generalizability, as well as criterion, convergent, concurrent, and predictive validity, as presented below. Moderation analyses were conducted using structural equation modeling in IBM SPSS Amos 28, and for significant interactions, simple slopes analysis was conducted using the Preacher et al. (2006) simple intercepts, simple slopes, and regions of significance in MLR 2-way interactions utility. Power analyses were conducted in G*Power 3.1.9.7 (Faul et al., 2007) to determine the necessary sample size to detect a significant moderation effect at .8 power given a model with two covariates plus the independent variable (neglect histories), moderator (resolution) and interaction term. Results indicated that for a small, medium, and large moderation effects respectively, sample sizes of 395, 55, and 25 participating mothers would be required. Thus, the present study was adequately powered to detect a medium or large moderation effect in all models except for Model 6, which examined moderation in the subsample of non-maltreating mothers only (*n* = 52). Additionally, bivariate correlations among key study variables are presented in Table 1.

### Evaluation of the maternal trauma resolution measure

The first aim of the study was to quantitatively evaluate the properties of the maternal childhood maltreatment trauma resolution code including reliability, variability, generalizability, validity, and predictive power of the resolution code relative to an existing self-report measure of the same construct (the PAAS Balancing-Forgiving subscale). To assess reliability, a random subsample (*n* = 44, 21.4%) of Parent Development Interviews were coded by the first author and two trained research assistants. Resulting interrater reliability was excellent (*ICC* = .937). Of the possible range of ratings, the full 1–9 scale was used, resulting in a mean resolution score of 6.04 (*SD* = 2.38). Next, associations between the resolution code and demographic variables were evaluated to assess generalizability. Bivariate correlations indicated that resolution scores were not significantly correlated with demographic factors, as shown in Table 2. Regarding categorical demographic variables, we also evaluated group differences in resolution scores by race, ethnicity, and marital status (Table 3). Using a One-Way ANOVA, there were no significant differences in resolution scores by racial group, (*F*(2, 195) = .681, *p* = .507) or by marital status (*F*(2, 195) = 1.453, *p* = .236). Using an independent samples *t*-test, there were no significant differences by ethnicity (e.g., Latinx vs. non-Latinx; *t*(196) = .080, *p* = .936). Given the use of narrative coding techniques in extracting this data, associations between the resolution scores and participants’ verbal ability were examined. Both vocabulary and comprehension scores on the WAIS-III were moderately correlated with resolution scores (*r* = .178; *r* = .220; See Table 2). Based on this finding, as well as best practice recommendations for narrative measures, applied analyses in this study controlled for verbal ability.

As part of the design of the larger RCT study, participating mothers were classified as either maltreating or non-maltreating controls based on coding of records with the Department of Human Services using the Maltreatment Classification System (MCS; Barnett et al., 1993). An independent samples *t*-test indicated that maltreating mothers (*M* = 5.75, *SD* = 2.43) had significantly lower resolution scores than non-maltreating mothers (*M* = 6.78, *SD* = 2.18), *t*(176) = 2.576, *p* =.011; See Table 4). These results provide preliminary evidence for criterion validity of the resolution measure.

Tests of convergent validity indicated that resolution scores and Balancing-Forgiving subscale scores were not correlated (*r* = .126, *p* = .108). Additionally, partial correlations controlling for maternal verbal comprehension and vocabulary also demonstrate weak correlations between resolution and Balancing-Forgiving scores (*r* = .108, *p* = .183), suggesting that verbal ability does not account for the failure of convergence between resolution and Balancing-Forgiving scores.

To examine concurrent validity, a series of stepwise linear regressions were conducted using the full sample. Specifically, we examine the relative predictive power of Resolution and Balancing-Forgiving scores for three theoretically relevant parenting constructs: parenting stress, parenting competence, and sensitivity. In Step 1, all control variables were entered, including maternal age and education, as well as vocabulary and verbal comprehension scores on the WAIS. In Step 2, Balancing-Forgiving scores were entered and in Step 3 resolution scores were entered. The first analysis examined parenting stress as the dependent variable. Results are presented in Table 5. Overall, Model 1 explained 9.6% of the variance in parenting stress, *R*^2^ = .096. The unique effects of Balancing-Forgiving scores on parenting stress were negligible, explaining less than 1% of variance (*sr^2^* < .001, *p* = .857). In contrast, resolution scores uniquely explained 2.1% of the variance in parenting stress when controlling for all other variables (*sr^2^* = .021, *p* = .044), such that higher resolution was associated with lower parenting stress (*B* = −2.565, *β* = −.150). We next examined parenting sense of competence as the dependent variable (Table 5). Overall, Model 2 explained 8.5% of the variance in parenting competence, *R*^2^ = .085. The unique effects of Balancing-Forgiving scores on parenting competence were negligible, explaining less than 1% of variance (*sr^2^* = .004, *p* = .409). In contrast, resolution scores uniquely explained 3.9% of the variance in parenting competence when controlling for all other variables (*sr^2^* = .039, *p* = .007), such that higher resolution was associated with higher parenting competence (*B* = .542, *β* = .204). Next, we examined observed maternal sensitivity at T1 as the dependent variable (Table 6). Model 3 explained 18.2% of the variance in maternal sensitivity, *R^2^* = .182. The unique effects of Balancing-Forgiving scores on sensitivity negligible (*sr^2^* = .002, *p* = .572). However, resolution scores uniquely explained 5.2% of variance in sensitivity (*sr^2^* = .052, *p* = .003), such that higher resolution was associated with higher observed sensitivity (*B* = .035, *β* = .239). Taken together, the results from this series of regressions suggests that Balancing-Forgiving scores were not related to parenting outcomes, whereas maternal childhood maltreatment trauma resolution scores were significantly associated with both reduced parenting stress and increased parenting competence and observed sensitivity at the same timepoint.

Our final set of validation analyses tested predictive validity through a series of stepwise regressions examining the contributions of resolution and Balancing-Forgiving scores on maternal psychopathology longitudinally. These analyses included only the subsample of mothers in the non-treatment groups (*n* = 132) to minimize confounding effects of the intervention on results. In Step 1, we entered all control variables, including maternal age, education, and verbal ability. In Step 2, we entered Balancing-Forgiving scores. In Step 3, we entered resolution scores. Results are presented in Table 7. Our first predictive validity regression model examined effects on maternal depression. Overall, Model 4 explained 10.8% of the variance in maternal depression symptoms, *R*^2^ = .108. The unique effect of Balancing-Forgiving scores on maternal depression symptoms was negligible, explaining less than 1% of variance (*sr^2^* = .001, *p* = .760). In contrast, resolution scores uniquely explained 9.9% of the variance in maternal depression when controlling for all other variables (*sr^2^* = .099, *p* = .004), such that higher resolution was associated with lower depression symptoms (*B* = −1.42, *β* = −.335). Our next analysis examined predictive effects on maternal anxiety over time. Model 5 explained 13.6% of the variance in maternal anxiety, *R^2^* = .136. Balancing-Forgiving scores uniquely explained less than 1% of the variance in maternal anxiety symptoms (*sr^2^* < .001, *p* = .927). In contrast, resolution was shown to be a robust predictor of maternal anxiety, accounting for 12.4% of the variance in maternal anxiety symptoms (*sr^2^* = .124, *p* = .001). These effects were again protective, such that higher resolution predicted lower maternal anxiety (*B* = −1.85, *β* = −.375).

### Buffering effects of childhood maltreatment trauma resolution on sequelae of maternal neglect histories

**Figure 3.**
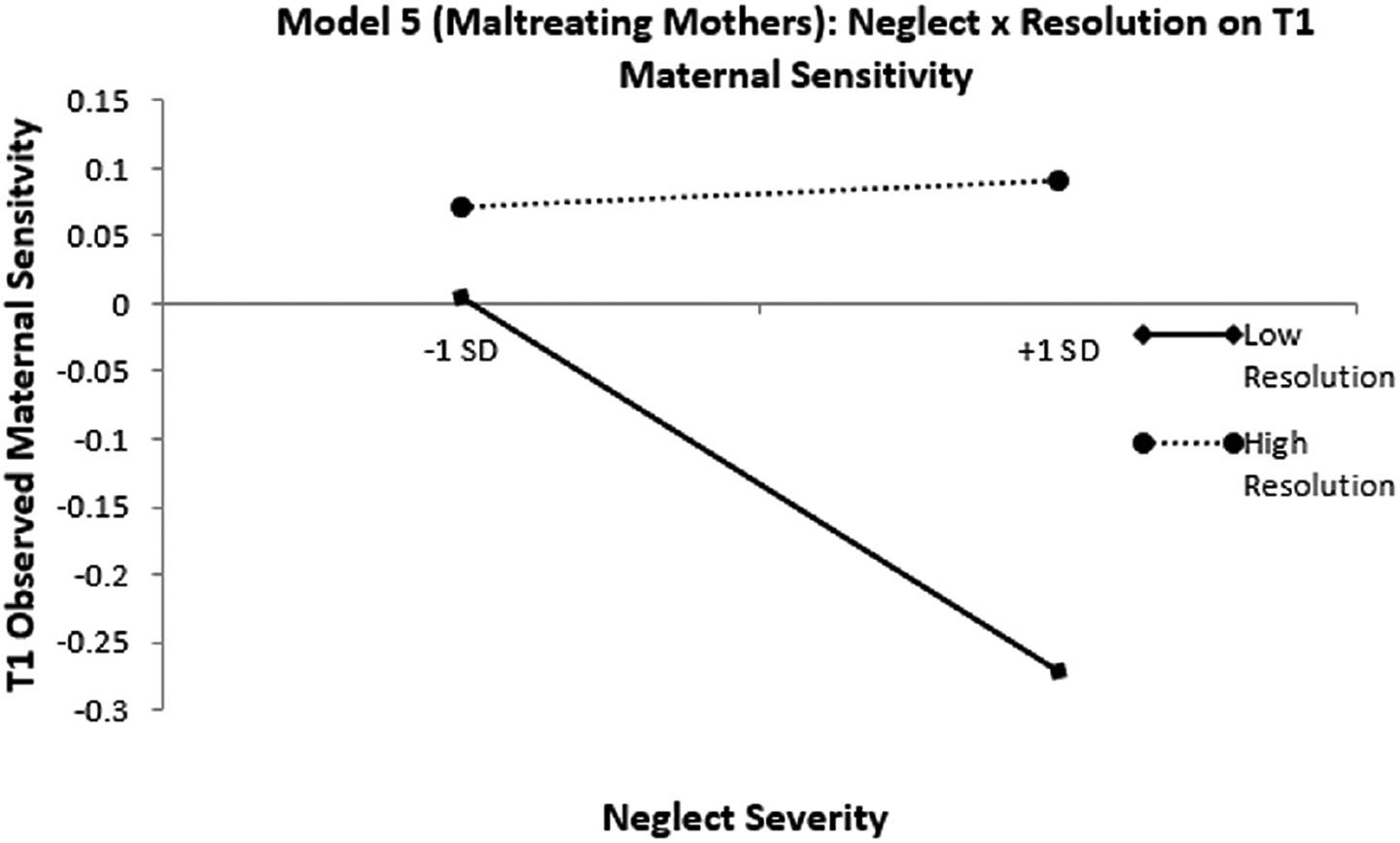
Simple slopes for cross-sectional moderation by maltreatment resolution in the maltreating subsample. Simple slopes plot of the cross-sectional effects of neglect severity on observed maternal resolution at high and low levels of trauma resolution among maltreating mothers only. Dotted line = nonsignificant slope. Solid line = significant slope. Results are indicative of protective-stabilizing.

Our final analysis tested maternal resolution as a moderator of associations between maternal neglect histories and parenting sensitivity (Aim 2). Model 6 tested the buffering hypothesis among maltreating mothers (*n* = 137) and Model 7 tested the buffering hypothesis among non-maltreating mothers (*n* = 52) assessed using a cross-sectional design. Model 8 tested moderation longitudinally in both maltreating and non-maltreating mothers combined (*n* = 132). To avoid confounding effects of treatment, only mothers who did not engage in treatment were included in longitudinal analyses. Prior to analyses, both trauma resolution and maternal neglect scores were mean-centered and a neglect history × trauma resolution interaction term was created by using the cross-product of the mean-centered neglect and resolution variables. Maternal age and verbal ability were added as covariates. The structural equation model was fully saturated, and thus model fit statistics are not presented. In maltreating mothers (Model 6), maternal neglect histories did not significantly predict maternal sensitivity at the same timepoint (*B* = −.078, *p* = .101). However, maternal trauma resolution did significantly predict maternal sensitivity (*B* = .044, *p* < .001). Likewise, the interaction of maternal trauma resolution and neglect histories significantly predicted maternal sensitivity (*B* = .037, *p* = .010). See Table 8 for all standardized and unstandardized regression weights. Based on this significant interaction term, we further probed the moderating role of maternal trauma resolution on maternal sensitivity using the Preacher et al., (2006) interactions utility. See Figure 3 for a graphical presentation of the simple slopes plots. The simple slope of the trendline plotting associations between maternal neglect histories and maternal sensitivity was statistically significant at −1 SD below the mean on maternal resolution (simple slope = −.168 (.059), *t* = −2.8459, *p* = .005), such that more severe histories of neglect were associated with lower maternal sensitivity at low levels of resolution. However, no such association was found at high levels of maternal trauma resolution (simple slope = .012 (.059), *t* = 0.202, *p* = .840). These results suggest maltreatment trauma resolution may act in a protective manner among maltreating mothers.

To examine whether maltreatment trauma resolution may operate differently among subgroups of participants with varying levels of risk, we also examined this moderation model among non-maltreating mothers only. Among these non-maltreating mothers (Model 7), maternal neglect histories did not significantly predict maternal sensitivity at the same timepoint (*B* = −.074, *p* = .265). Maternal trauma resolution did not significantly predict maternal sensitivity among non-maltreating mothers either (*B* = −.012, *p* = .574). Finally, the interaction of maternal trauma resolution and neglect histories did not significantly predict maternal sensitivity (*B* = .016, *p* = .418). See Table 8 for all regression weights.

**Figure 4.**
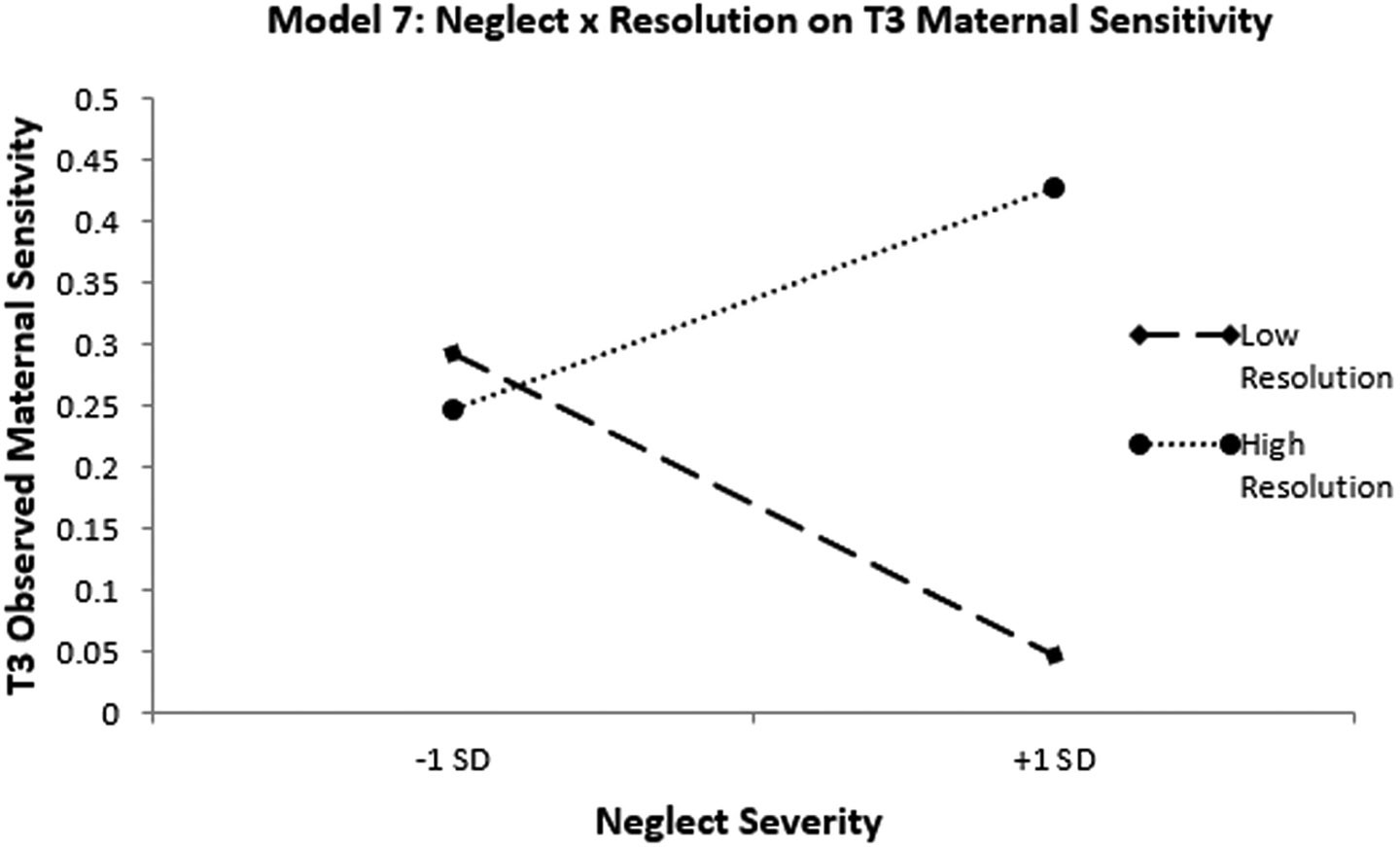
Simple slopes for longitudinal moderation by maltreatment resolution. Simple slopes plot of the longitudinal effects of neglect severity on observed maternal resolution at high and low levels of trauma resolution in the non-treatment groups. Dotted line = nonsignificant slope. Results are indicative of protective-enhancing.

Finally, we also tested a longitudinal moderation model among the non-treatment groups of the sample, which included both maltreating and non-maltreating mothers (Model 8). All standardized and unstandardized regression weights are presented in Table 9. Controlling for maternal age and verbal ability, maternal neglect histories did not significantly predict maternal sensitivity at T3 (*B* = −.019, *p* = .721). Maternal trauma resolution at T1 marginally predicted maternal sensitivity at T3 (*B* = .035, *p* = .059). The interaction of maternal trauma resolution and neglect histories significantly predicted maternal sensitivity at T3 (*B* = .051, *p* = .005). Based on this significant interaction term, we further probed the moderating role of maternal maltreatment trauma resolution on maternal sensitivity. The slope of the trendline plotting associations between maternal neglect histories and maternal sensitivity was not significant at either −1 SD below the mean on maternal resolution (simple slope = −.142 (.114), *t* = −1.244, *p* = .216) or +1 SD above the mean (simple slope = .104 (.114), *t* = .911, *p* = 0.364). Although the slope of the trend lines did not reach significance, visual inspection of the plotted simple slopes reveals interesting differences in the association between neglect histories and maternal sensitivity at high vs. low resolution that suggest a protective-enhancing effect of resolution (See Figure 4).

## Discussion

The current study adds to the literature on intergenerational transmission of maltreatment in several important ways. First, the results of this study address key gaps in the literature by 1) identifying and developing a measure of a potentially powerful protective factor against intergenerational transmission of maladaptive caregiving for mothers with maltreatment histories and 2) investigating the protective effects of resolution for mothers with neglect histories. This second contribution is particularly significant, as research on neglect has lagged behind research on other forms of maltreatment (Mulder et al., 2018; Stoltenborgh et al., 2013). Additionally, we present both cross-sectional and longitudinal findings, allowing for a small degree of temporal inference in our conclusions. The present study further utilized a creative, multimethod design for assessing key constructs of interest that included self-report, narrative, and observational measures. The inclusion of multimethod assessments reduces shared method variance and enhances interpretability of findings. In particular, using observational assessments of maternal sensitivity is a noteworthy strength. Research suggests that high parent distress and low family socioeconomic status are linked to lower agreement between self-report and observational measures of parenting (Herbers et al., 2017). As maltreating samples may have elevated rates of both distress and financial hardship, inclusion of observational assessments is important for accurate assessment, particularly for CPS-impacted parents who may be hesitant to self-report difficulties or perceived weaknesses in their parenting. Finally, the sample includes diverse families from low-income backgrounds living in elevated ecological distress. Thus, the present study helps identify protective factors for a key population of interest.

Results presented here suggest that the maternal trauma resolution measure has strong reliability. Additionally, resolution scores were not significantly associated with any key demographic variables. This is promising for generalizability, although we believe additional replication with diverse samples, including more socioeconomically stratified samples, would further bolster generalizability. However, when examining convergent validity, the maltreatment trauma resolution code scores and Balancing-Forgiving subscale scores were not significantly correlated, even after controlling for verbal ability. While this failure to demonstrate convergent validity between the resolution measure and the Balancing-Forgiving subscale of the PAAS may be interpreted as indicating that the resolution code has poor validity, it is also possible that the low convergence may instead indicate that the Balancing-Forgiving subscale of the PAAS does not adequately assess this construct.

Supporting this interpretation, other subscales of the PAAS have been used in existing research more widely (e.g., Huth-Bocks et al., 2004; Newman et al., 2015), whereas the Balancing-Forgiving subscale is used relatively infrequently in published research. This lack of uptake is puzzling given that prior research emphasizes the need to characterize protective, buffering processes in the aftermath of maltreatment and relational trauma (Dixon et al., 2009; Toth & Cicchetti, 2013). In those studies that did include the Balancing-Forgiving subscale (e.g., Cassidy et al., 2009; Cicchetti et al., 2006), significant findings were reported for other subscales of the PAAS, but not for the Balancing-Forgiving subscale. In both of these studies, the internal consistency of the Balancing-Forgiving subscale was also lower than other subscales of the PAAS, as was the case in our sample (αs = .651–.70). These somewhat lower internal consistencies relative to other subscales of the PAAS may partially explain the lack of uptake for the Balancing-Forgiving subscale in the extant literature, as such low alphas may attenuate the strengths of associations between the Balancing-Forgiving subscale and other outcome variables, making researchers less likely to publish findings using this scale.

We further argue that self-report measures may not be methodologically well-suited to assessing the construct of resolution. Anecdotally, many mothers in this sample initially provided positive, surface-level descriptions of their own mothers during the Parent Development Interview, but either were unable to elaborate on or even later contradicted these initial positive descriptions when probed further by interviewers. We believe that these mothers may report high levels of forgiveness and resolution on self-report measures such as the PAAS either due to trauma-related biases in reporting such as avoidance/suppression, or demand characteristics such as social desirability. In contrast, administration of the Parent Development Interview involves rapport building and follow-up probing that may yield more nuanced information about the adult parent-child relationship. Thus, resolution scores coded from the Parent Development Interview may be better able to tap into this notion of forgiveness and acceptance on a deeper, implicit level by evaluating elaboration and consistency of participants’ descriptions of their adult mother–child relationships. This interpretation is supported by findings from Models 1–5 in the current study, which found that the resolution code consistently outperformed the Balancing-Forgiving subscale when predicting theoretically relevant outcomes, including parenting stress, parenting sense of competence, sensitivity, and maternal psychopathology. Moreover, this pattern of findings was found both cross-sectionally in the full sample and longitudinally in the non-treatment groups subsample.

Other dimensional coding systems have been developed to assess constructs that may fall within the broader nomological net of concepts potentially linked with resolution of childhood maltreatment trauma, such as reflective functioning and angel memories. We suggest future research may examine resolution, reflective functioning, and angel memories concurrently to better understand how these potentially interrelated capacities may support caregiving in unique and overlapping ways. Reflective functioning refers to the capacity to use underlying mental states and intentions to understand behavior (Luyten et al., 2017; Slade et al., 2004; Sleed et al., 2020). Within the caregiving domain, reflective functioning may support sensitive parenting and development of quality attachment relationships (see Camoirano, 2017 for a review). Prior work (e.g., Borelli et al., 2019; Ensink et al., 2015) suggests mentalizing about trauma-related content may require particularly strong reflective functioning capacities. For example, previous research suggests trauma-focused reflective functioning may serve as a buffer against intergenerational transmission of child sexual abuse (Borelli et al., 2019). In terms of links to resolution as assessed in the current study, broader capacities for reflective functioning such as perspective-taking and awareness of one’s own emotions could support mothers in resolving feelings and relational issues associated with their own traumatic caregiving experiences growing up (Ensink et al., 2014).

Another key internal resource that may disrupt intergenerational transmission of maltreatment concerns “angel memories.” Grounded in psychodynamic theories of psychic defenses, Angels in the Nursery frameworks propose that the ability to clearly and coherently recount benevolent childhood memories may support the ability to re-create positive caregiving moments with offspring (Narayan et al., 2019). Importantly, these angel memories are hypothesized to act as a powerful internal resource even if these benevolent childhood experiences were infrequent or accompanied by traumatic or abusive experiences in childhood. In support of this protective effect, maternal angel memories have been shown to buffer links between maternal childhood maltreatment histories and adult PTSD symptoms (Narayan et al., 2017) and offspring trauma exposure (Narayan et al., 2019). The ability to draw on angel memories may support resolution of childhood maltreatment trauma by elevating the salience of positive aspects of challenging and complicated family relationships and reducing maladaptive psychic defenses, such as black and white thinking about caregivers.

After presenting results in support of the reliability and validity of the resolution code, we leveraged this novel measure to evaluate the potential buffering effects of maternal trauma resolution on reductions in parenting sensitivity following exposure to neglect in childhood. Analyses tested this pattern of moderation cross-sectionally in maltreating and non-maltreating mothers respectively. In maltreating mothers, mothers with high resolution demonstrated consistently sensitive parenting regardless of the amount of neglect experienced during childhood, whereas in mothers with low resolution, neglect histories were significantly associated with reduced parenting sensitivity. This is indicative of a protective-stabilizing model of resilience for resolution (Luthar et al., 2003). However, no pattern of moderation was found in the non-maltreating subsample. This may suggest that resolution operates as a protective factor only under conditions of higher risk and distress, but further replication is needed to affirm this interpretation, particularly in lower distress samples.

We also evaluated the same moderation model longitudinally in the non-treatment groups, collapsing maltreating and non-maltreating mothers to increase statistical power. In this model, a protective effect of high maternal resolution was again found, but this pattern of moderation was more consistent with a protective-enhancing model of resilience (Luthar et al., 2003). That is, maternal neglect histories were modestly associated with more sensitive parenting at higher levels of neglect in mothers with high trauma resolution, whereas in mothers with low trauma resolution, neglect histories were modestly associated with lower maternal sensitivity. However, neither of the trend lines’ slopes reached statistical significance. Protective-enhancing patterns of resilience suggest that the ability to engage and cope with stressors may actually enhance functioning, or in this case, enhance maternal sensitivity. Such a model would suggest that maternal trauma resolution may be a powerful clinical target that could facilitate increased maternal sensitivity. Moreover, from a basic research perspective, these findings follow recommendations from previous reviews (e.g., Cicchetti, 2013) to characterize dynamic developmental processes that may facilitate resilience following maltreatment.

The results of the present study are tempered by several limitations. The measure used to test convergent validity, the Balancing-Forgiving subscale of the PAAS, had low internal consistency in this sample. Thus, future research may seek to demonstrate convergence between the resolution code and more reliable measures of similar constructs, such as coding systems for the Adult Attachment Interview or various measures of reflective functioning. Additionally, while this sample was racially and ethnically diverse, it was restricted to families from low-income backgrounds, limiting generalizability of findings to higher SES groups. This study also focused exclusively on mothers, leaving the role of maltreatment trauma resolution in fathers undescribed. Although the current coding scheme emphasizes the degree of forgiveness and acceptance in the current relationship between adult offspring and their caregivers, current feelings of forgiveness and acceptance often concerned past events and transgressions that occurred during childhood. Thus, although the emphasis was on current feelings of acceptance and forgiveness, depression-related biases and other limitations associated with retrospective report may still apply to the Resolution measure. Finally, we chose to focus exclusively on the intergenerational effects of neglect. While this choice was intentional based on the prevalence of neglect and lack of research in this area, it is also worth acknowledging that maltreatment subtypes often co-occur, and complex heterogeneity in maltreatment experiences across development often complicates the study of maltreatment subtypes (Warmingham et al., 2019). Future research should evaluate the protective effects of resolution across maltreatment subtypes.

Despite these limitations, the present study makes significant contributions to the extant literature. First, the results of this study support the reliability and validity of a novel measure of maternal childhood maltreatment trauma resolution. Thus, the development and dissemination of this coding scheme adds an additional tool for assessing clinical processes to developmental psychopathologists’ methodological arsenal. Second, results from moderation models suggest that maternal trauma resolution may play a protective role in ameliorating deficits in sensitive parenting following exposure to childhood neglect. Thus, the present research can be used to inform theoretical and psychodynamic perspectives on resilience. Finally, the present research possesses translational implications for clinical practice with distressed families. For example, strategies for targeting maternal maltreatment trauma resolution may be incorporated into existing trauma-informed therapies. Additionally, clinicians may seek to utilize measures of maltreatment trauma resolution into their practice to assess patient progress. Despite these significant contributions, we also believe that additional research is warranted, and conclude with several recommendations.

While the present results collectively suggest that maternal childhood maltreatment trauma resolution may operate as a protective factor, it is not clear from these results whether resolution operates as a protective-stabilizing or protective-enhancing factor. Thus, replication studies are needed. If maltreatment trauma resolution indeed acts as a protective-enhancing factor as suggested by the longitudinal portion of these results, then high maternal resolution may enhance maternal sensitivity through a number of potential mechanisms, such as enhancing maternal identity construction and identity clarity, bolstering interpersonal relationships, and encouraging perspective-taking and more nuanced thinking about complicated relationships. Assessing resolution of child maltreatment trauma alongside theoretically related constructs such as trauma-focused reflective functioning (e.g., Ensink et al., 2015) and angel memories (e.g., Narayan et al., 2019) could help clarify the shared and distinct contributions of internal coping resources as protective factors.

Additionally, further replication is needed in larger samples of non-maltreating mothers to clarify whether resolution may operate in a protective manner across the general population, or only in highly distressed, at-risk individuals. Larger, more representative samples may also be used to establish meaningful cut-points on the resolution measure to be used as a screening tool for risk for maltreatment perpetration. Ideally, these studies could assess resolution prenatally to establish risk level prior to the occurrence of maltreatment. Future studies should also include other caregivers, such as fathers. Finally, researchers may also seek to include the present measure of maternal trauma resolution in treatment studies. Kazdin (2007) argues that while research on evidence-based psychotherapy has proliferated in recent decades, studies of treatment *mechanisms* are still desperately lacking. For example, while researchers have theorized treatment mechanisms for interventions such as Child-Parent Psychotherapy (Lieberman et al., 2006; Lieberman, 2004), researchers are still in the early stages of formally empirically testing proposed treatment mechanisms of this intervention (Alto et al., 2021; Cerulli et al., 2021; Toth et al., 2021). Consequently, future treatment mechanism studies of the protective effects of maternal trauma resolution can inform clinical practice and the design of future interventions, and ultimately be leveraged to reduce the negative intergenerational effects of maltreatment on future generations.

## Supplementary Material

Supplements

## Figures and Tables

**Table 1.** Correlations for key study variables

Variable	1	2	3	4	5	6	7	8	9
1. Maltreatment trauma resolution	–								
2. Balancing-forgiving (PAAS)	.126	–							
3. Physical neglect history (CTQ)	−.375**	−.332**	–						
4. Parenting stress (PSI)	−.195**	−.068	.336**	–					
5. Parenting competence (PSI)	−.190**	−.107	.270**	.777**	–				
6. Depression (SCL-90-R)	−.225**	.045	.293**	.457**	.398**	–			
7. Anxiety (SCL-90-R)	−.228**	.004	.226**	.415**	.397**	.745**	–		
8. T1 Maternal sensitivity (MBQ)	.326**	.069	−.191**	−.210**	−.120	−.073	−.108	–	
9. T3 Maternal sensitivity (MBQ)	.208*	.160	−.202*	−.175*	−.141	−.081	−.164	.595**	–

*Note.* *p < .05, **p < .01. Bivariate correlations between key study variables are presented above.

**Table 2.** Correlation matrix for resolution scores and demographic variables

Variable	1	2	3	4	5	6	7
1. Resolution	–						
2. Maternal age	**.084**	–					
3. Total income	**.010**	.087	–				
4. Public assistance	**−.044**	.135	.222**	–			
5. Highest education level	**.120**	.190**	.134	−.148*	–		
6. Occupational prestige score	**.080**	−.062	.334**	−.280**	.287**	–	
7. Vocabulary (WAIS-III)	**.178** *	−.017	.038	−.124	.427**	.134	–
8. Comprehension (WAIS-III)	**.220** **	−.086	.025	−.080	.432**	.137	.673**

*Note.* Bivariate correlations are presented above. Total Income and Amount Public Assistance Received were measured in thousands of dollars USD. *p < .05, **p < .01.

**Table 3.** Resolution score group means and standard deviations by race, ethnicity and marital status

	*n*	*M*	SD	Min	Max
Race					
Black or African American	106	6.21	2.30	1	9
White	50	5.96	2.62	1	9
Multiracial or other	42	5.71	2.28	1	9
Ethnicity					
Latinx	20	6.00	2.37	1	9
Non-Latinx	178	6.04	2.58	1	9
Marital status					
Married	22	5.32	2.17	1	9
Living together	33	6.42	2.67	1	9
Single	143	6.06	2.34	1	9

*Note. M* = mean, SD = standard deviation. Min = minimum. Max = maximum. There were no significant differences in resolution scores by racial group, *F*(2, 195) = .681, *p* = .507, by marital status *F*(2, 195) = 1.453, *p* = .236, or ethnicity (e.g., Latinx vs. non-Latinx), *t*(196) = .080, *p* = .936.

**Table 4.** Resolution score group means by maltreating status

	*n*	*M*	SD	Min	Max
Maltreating status					
Maltreating mothers	129	5.75	2.43	1	9
Non-maltreating mothers	49	6.78	2.18	1	9

*Note. M* = mean, SD = standard deviation. Min = minimum. Max = maximum. Resolution scores were significantly lower in maltreating mothers compared to non-maltreating mothers, *t*(176) = 2.576, *p* = .011.

**Table 5.** Regression models 1 & 2: Evaluating concurrent validity for parenting stress and parenting competence (maternal self-report)

	*B* (SE)	*β*	*t*	*p*	sr^2^
Model 1 (Parenting stress)					
*Model 1 Step 1*					
Maternal age	−1.107 (.499)	−.164	−2.217	.028*	.026
Maternal education	−3.753 (3.732)	−.083	−1.006	.316	.005
Vocabulary (WAIS-III)	−1.363 (1.499)	−.091	−909	.364	.004
Verbal comprehension (WAIS-III)	−1.255 (1.403)	−.090	−.895	.372	.004
*Model 1 Step 2*					
Maternal age	−1.087 (.509)	−.161	−2.137	.034*	.024
Maternal education	−3.732 (3.743)	−.083	−.997	.320	.005
Vocabulary (WAIS-III)	−1.366 (1.503)	−.091	−.909	.365	.004
Verbal comprehension (WAIS-III)	−1.265 (1.408)	−.091	−.899	.370	.004
Balancing-forgiving (PAAS)	.000 (.001)	−.017	−.229	.819	<.001
*Model 1 Step 3*					
Maternal age	−.991 (.506)	−.147	−1.956	.052†	.020
Maternal education	−3.732 (3.711)	−.083	−1.006	.316	.005
Vocabulary (WAIS-III)	−1.162 (1.493)	−.078	−.778	.437	.003
Verbal comprehension (WAIS-III)	−.935 (1.405)	−.067	−.665	.507	.002
Balancing-forgiving (PAAS)	.000 (.001)	−.017	−.181	.857	<.001
Resolution	−2.565 (1.266)	−.150	−2.026	.044*	.021
Model 2 (Parenting competence)					
*Model 2 Step 1*					
Maternal age	.109 (.079)	.104	1.385	.168	.010
Maternal education	1.004 (.589)	.143	1.705	.090†	.016
Vocabulary (WAIS-III)	−.362 (.236)	−.156	−1.532	.127	.013
Verbal comprehension (WAIS-III)	.231 (.221)	.107	1.043	.298	.006
*Model 2 Step 2*					
Maternal age	.097 (.080)	.092	1.209	.228	.008
Maternal education	.992 (.589)	.141	1.682	.094†	.015
Vocabulary (WAIS-III)	−.360 (.237)	−.155	−1.523	.130	.013
Verbal comprehension (WAIS-III)	.237 (.222)	.110	1.068	.287	.006
Balancing-forgiving (PAAS)	.000 (.000)	.066	.880	.380	.004
*Model 2 Step 3*					
Maternal age	.076 (.079)	.073	.968	.334	.005
Maternal education	.991 (.579)	.141	1.713	.088†	.028
Vocabulary (WAIS-III)	−.403 (.233)	−.174	−1.732	.085†	.031
Verbal comprehension (WAIS-III)	.167 (.219)	.078	.762	.447	.003
Balancing-forgiving (PAAS)	.000 (.000)	.061	.827	.409	.002
Resolution	.542 (.197)	.204	2.744	.007**	.039

*Note.* Unstandardized and standardized regression weights for parenting stress and parenting sense of competence models. †*p* < .10, *p < .05, **p < .01.

**Table 6.** Regression model 3: Evaluating concurrent validity for T1 maternal sensitivity (observed)

	*B* (SE)	β	*t*	*p*	sr^2^
Model 3 (Maternal sensitivity)					
*Model 3 Step 1*					
Maternal age	.002 (.005)	.028	.342	.733	<.001
Maternal education	.052 (.035)	.135	1.465	.145	.013
Vocabulary (WAIS-III)	.028 (.014)	.217	1.922	.057†	.023
Verbal comprehension (WAIS-III)	.008 (.013)	.066	.575	.567	.002
*Model 3 Step 2*					
Maternal age	.002 (.005)	.026	.322	.748	<.001
Maternal education	.056 (.036)	.146	1.569	.119	.015
Vocabulary (WAIS-III)	.028 (.014)	.222	1.956	.052†	.023
Verbal comprehension (WAIS-III)	.006 (.013)	.053	.459	.647	.001
Balancing-forgiving (PAAS)	.041 (.045)	.073	.919	.360	.005
*Model 3 Step 3*					
Maternal age	.000 (.005)	−.003	−.036	.971	<.001
Maternal education	.055 (.035)	.143	1.578	.117	<.001
Vocabulary (WAIS-III)	.025 (.014)	.194	1.752	.082†	.015
Verbal comprehension (WAIS-III)	.002 (.013)	.019	.166	.868	<.001
Balancing-forgiving (PAAS)	.025 (.044)	.044	.566	.572	.002
Resolution	.035 (.012)	.239	2.980	.003**	.052

*Note.* Unstandardized and standardized regression weights for the observed maternal sensitivity model. †*p* < .10, *p < .05, **p < .01.

**Table 7.** Regression models 4 & 5: Evaluating predictive validity for maternal psychopathology outcomes

	*B* (SE)	*β*	*t*	*p*	sr^2^
Model 4 (Depression)					
*Model 4 Step 1*					
Maternal age	.019 (.219)	.010	.088	.930	<.001
Maternal education	−.210 (1.508)	−.019	−.139	.890	<.001
Vocabulary (WAIS-III)	−.322 (.545)	−.089	−.591	.556	.004
Verbal comprehension (WAIS-III)	.011 (.514)	.003	.021	.983	<.001
*Model 4 Step 2*					
Maternal age	.019 (.220)	.010	.087	.931	<.001
Maternal education	−.211 (1.527)	−.019	−.138	.891	<.001
Vocabulary (WAIS-III)	−.322 (.549)	−.089	−.588	.558	.004
Verbal comprehension (WAIS-III)	.011 (.521)	.003	.021	.983	<.001
Balancing-forgiving (PAAS)	−.007 (1.919)	.000	−.004	.997	<.001
*Model 4 Step 3*					
Maternal age	.131 (.214)	.068	.616	.540	.004
Maternal education	−.040 (1.46)	−.004	−.027	.978	<.001
Vocabulary (WAIS-III)	−.287 (.524)	−.079	−.548	.585	.003
Verbal comprehension (WAIS-III)	.293 (.507)	.086	.578	.565	.004
Balancing-forgiving (PAAS)	.566 (1.843)	.033	.307	.760	.001
Resolution	−1.417 (.483)	−.335	−2.934	.004*	.099
Model 5 (Anxiety)					
*Model 5 Step 1*					
Maternal age	−.181 (.254)	−.081	−.713	.478	.006
Maternal education	.352 (1.751)	.027	.201	.841	<.001
Vocabulary (WAIS-III)	−.418 (.633)	−.099	−.661	.511	.005
Verbal comprehension (WAIS-III)	.122 (.597)	.031	.205	.838	<.001
*Model 5 Step 2*					
Maternal age	−.179 (.255)	−.080	−.701	.485	.006
Maternal education	.304 (1.772)	.023	.171	.864	<.001
Vocabulary (WAIS-III)	−.420 (.637)	−.099	−.659	.512	.005
Verbal comprehension (WAIS-III)	.141 (.605)	.035	.233	.816	<.001
Balancing-forgiving (PAAS)	−.553 (2.228)	−.028	−.248	.805	<.001
*Model 5 Step 3*					
Maternal age	−.033 (.244)	−.015	−.134	.894	<.001
Maternal education	.526 (1.670)	.040	.315	.754	.001
Vocabulary (WAIS-III)	−.374 (.600)	−.089	−.624	.535	.004
Verbal comprehension (WAIS-III)	.508 (.580)	.128	.876	.384	.008
Balancing-forgiving (PAAS)	.194 (.109)	.010	.092	.927	<.001
Resolution	−1.845 (.552)	−.375	−3.340	.001*	.124

*Note.* Unstandardized and standardized regression weights for maternal depression and anxiety models. †*p* < .10, *p < .05, **p < .01.

**Table 8.** Unstandardized and standardized SEM regression weights for cross-sectional moderation by resolution in maltreating subsample (model 6) and non-maltreating subsample (model 7)

Model 6: Maltreating subsample	*B*	SE	*β*	*p*
Maternal age → T1 Sensitivity	.001	.005	.026	.746
Verbal ability → T1 Sensitivity	.021	.011	.149	.063†
Neglect history → T1 Sensitivity	−.078	.048	−.178	.101
Resolution → T1 Sensitivity	.044	.013	.298	<.001***
Neglect × Resolution → T1 Sensitivity	.035	.011	.264	.010**
Model 7: Non-Maltreating Subsample	*B*	SE	*β*	*p*
Maternal age → T1 Sensitivity	.002	.008	.033	.816
Verbal ability → T1 Sensitivity	.032	.015	.305	.035*
Neglect history → T1 Sensitivity	−.074	.066	−.217	.265
Resolution → T1 Sensitivity	−.012	.022	−.094	.574
Neglect × Resolution → T1 Sensitivity	.016	.020	.176	.418

*Note.* †*p* < .10, *p < .05, **p < .01, ***p < .001.

**Table 9.** Unstandardized and standardized SEM regression weights for longitudinal moderation by resolution in non-treatment groups (model 8)

Model 8: Non-treatment groups	*B*	SE	*β*	*p*
Maternal age → T3 Sensitivity	−.009	.007	−.129	.164
Verbal ability → T3 Sensitivity	.040	.014	.264	.005**
Neglect history → T3 Sensitivity	−.019	.053	−.040	.721
Resolution → T3 Sensitivity	.035	.019	.204	.059†
Neglect × Resolution → T3 Sensitivity	.051	.018	.287	.005**

*Note.* †*p* < .10, *p < .05, **p < .01, ***p < .001. The non-treatment groups included both maltreating and non-maltreating mothers. Treatment groups were excluded from analyses to avoid confounding effects of the intervention in the larger study.
