# Supplementary material for: Maternal childhood maltreatment trauma resolution: Development of a novel narrative coding measure and implications for intergenerational parenting processes
Source: Dev Psychopathol. Author manuscript; Available in PMC 2025 Apr 27. (PMC12034303; doi:10.1017/S0954579423001256)
Supplement: Supplements [file NIHMS2073798-supplement-Supplements.docx]

**Supplemental Material**

This supplemental material includes (1) item content for the Perceptions of Adult Attachment Scale (PAAS); (2) a more detailed description of the Resolution code from the IIRCS coding manual; and (3) narrative excerpts corresponding to each Resolution anchor score.

*Please note, a full copy of the IIRCS manual, including the introduction/overview and other codes is available upon request. Please contact the first author.

**S1. Perceptions of Adult Attachment Scale-Modified (PAAS)**

Item Scale: 1. Strongly Disagree; 2. Disagree; 3. Neutral; 4. Agree; 5. Strongly Agree

Rejected/Neglected:

3. My mother was not very affectionate.

4. When I was a young child and little things went wrong, I did not feel sure I could count on my mother to take care of me.

18. In childhood I know I was low on my mother’s priority list.

29. In childhood I often had the impression that my mother was not listening to me. She often tuned me out.

45. If something really bad happened to me in childhood, I did not feel I could count on my mother to support me.

46. When I was a child I sometimes got the feeling that my mother wished I was never born.

Rejected/Threatened:

10. When I was a child my mother sometimes told me that if I was not good she would stop loving me.

23. In childhood my mother sometimes threatened to leave me or to send me away if I wasn’t good.

33. I remember my mother telling me that I didn’t pay enough attention to her or love her enough.

52. When I acted bad as a child my mother would at times threaten to send me away.

**Balancing-Forgiving:**

8. Neither my mother nor myself are perfect but somehow we made it through my childhood.

17. Even though I went through rough times with my mother during my childhood, somewhere along the line I managed to let go of the majority of those angry, hurt feelings.

25. Even with all our past difficulties, I realize my mother did the best for me that she could.

31. My mother and I are more accepting of each other’s differences than we have been in the past.

44. If my mother was not fair to me as a child I realize now it was because she was dealing with her own problems.

54. For all our past problems my mother and I can still enjoy a good laugh together.

60. When I think back to my early childhood experiences I discover things about myself and my parents that I’ve never consider before.

Angry:

11. My mother is selfishly caught up in herself to the exclusion of everybody else.

20. No one gets under my skin like my mother.

28. There are times when I feel like shaking my mother and saying, “Wake up and see me for who I am.”

35. My mother is a real nag.

**S2. Maltreatment Trauma Resolution Excerpt from Infant and Intergenerational Relationships Coding System Manual**

# Intergenerational Team Codes

**Resolution**

**Resolution:** This scale assesses the degree of acceptance and forgiveness presently held by the mother regarding the experiences and circumstances of her childhood upbringing.

This scale conceptualizes resolution as an internal process that unfolds gradually over time. Moreover, this process may be nonlinear and ebb and flow over time. The narratives reflect mothers’ current progress along the path to acceptance and forgiveness for negative past experiences and transgressions that may have occurred during her childhood. Evidence of advanced resolution includes perspective-taking, self-reflection, reassessing or recontextualizing past events, and integrating new information into her representations of her relationships. The forgiveness aspect of resolution in particular may include letting go of negative feelings towards the caregiver/transgressor, empathy for the caregiver and their circumstances, improved relationships or attempts to improve relationships with the caregiver, and other prosocial change towards the caregiver.

A complete absence of resolution may be marked by denial or avoidance of childhood memories, rumination or anger, or a generally disorganized representation of the caregiver and her childhood experiences. Somewhat lower ratings may be assigned to those mothers who have just entered the earlier stages of resolution, which may involve conscious awareness and acknowledgement of anger or negative feelings harbored towards her caregivers, even if she is unable to address these feelings at present. Mothers may also begin to show decreased motivation for retaliation, revenge, or estrangement from the perpetrator around this time. In making progress towards resolution and forgiveness, the mother may begin to accept the circumstances of her upbringing, and acknowledge the consequences and feelings that may arise as a result. The highest ratings of resolution should be assigned to those mothers who have initiated actions to heal themselves or repair relationships with caregivers, particularly if those efforts include prosocial actions towards the caregiver.

Notes:

The degree of relationship disruption and trauma experienced by mothers will vary. Themes of acceptance and forgiveness may be less apparent in the narratives of mothers who endorse more positive upbringings. However, even healthy and caring parent-child relationships involve ruptures and disagreements. Coders’ ratings should reflect the resolution of even these minor relationship ruptures.

Additionally, high resolution scores may still be assigned without initiation of prosocial behavior towards the caregiver, particularly if such prosocial behavior may endanger the mother or her infant.

**Resolution Ratings/Exemplars:**

**1) Not at all characteristic:** The narrative provides no evidence of acceptance or forgiveness for her childhood upbringing. The mother’s present state of mind may be marked by denial, avoidance or suppression, harbored anger, or the mother may report otherwise highly disorganized representations of her relationship with her caregiver.

**Examples:** The mother may not be able to provide a coherent account of her current relationship with caregiver or report that they currently have no relationship (e.g. are not on speaking terms). The mother may express a desire to retaliate against the caregiver/transgressor or “punish” the caregiver for past actions.

**2):** Between a 1 and 3.

**3) Minimally characteristic:** The narrative may include acknowledgement of negative feelings harbored towards the caregiver, but it seems the mother is largely unready to address these feelings.

**Examples:** The coder may be left with the impression that the mother is just beginning to grapple with these feelings. She may not wish any ill on the caregiver but she is not ready to forgive either.

**4):** Between a 3 and a 5.

**5) Somewhat characteristic:** The mother may acknowledge having mixed feelings about her upbringing and endorse a desire or willingness to work on unresolved feelings stemming from her upbringing, but struggle with how exactly to follow through on addressing her feelings.

**Examples:** The mother may openly discuss some of the ways that her childhood experiences impact her life today and express a desire to work on herself or her relationships. However, it does not seem she has taken concrete actions to work on these issues to date.

**6):** Between a 5 and 7.

**7) Moderately characteristic:** The mother is able to acknowledge the ways her upbringing affects her today and any feelings she currently holds regarding her relationship with the caregiver. She has **taken action to begin the healing process**, particularly efforts to work on herself (e.g. seeking social support/talking to others, seeking mental health treatment, engaging in self-reflection) and may be in the process of forgiving her caregiver. However, she may still be hesitant about initiating prosocial actions towards the caregiver.

**Examples:** The narrative shows that a degree of healing and reflection has taken place, driven by concrete actions and efforts to address her feelings about her relationship with the caregiver. However, the relationship between the mother and caregiver may still be hampered by past disruptions or transgressions.

**8):** Between a 7 and a 9.

**9) Mainly characteristic:** The mother reports taking prosocial actions towards the caregiver or making an effort to improve the relationship with the caregiver, or otherwise openly acknowledges acceptance towards the caregiver. Or, the mother may simply endorse an overall positive and open relationship with the caregiver.

**Examples:** Efforts to repair the relationship between the mother and her caregiver are evident or exceptional evidence of acceptance is noted. The relationship does not have to be completely positive at present but shows marked improvement.

**S3. Table of narrative excerpts and corresponding Resolution scores**

| **Resolution Score** | **Corresponding Narrative Excerpt** | **Notable features** |
| --- | --- | --- |
| 1 | “I can’t describe [my mother] because I’ve had… I was abused as a child. So… It’s a bad subject.” | -Inability to provide details, even after follow-up prompts  -Pauses indicate possible hesitancy to engage with emotional content  -Evidence of avoidance or suppression |
| 3 | “(Sigh) She always knew to beat me, she never knew how to say nothing nice. She thought she could buy me with clothes or toys. She always was yelling at me, she beat my face, she spit on my- on me, she hit me with a baseball bat, she broke up, she broke glass, plates. She burnt, burned my chest. She clawed my face with a cube of ice.(Sigh) She beat my ears, she beat everything that I think you only do to, not even to animals, you do those kind of stuff. My mother, I don’t know, she’s still my mother. I think I, you know, I don’t think, I know I love my mother but not like my grandmother.” | -Thinking about the caregiver evokes intensely negative memories, but the mother is able to name these traumatic experiences (acknowledgement of negative feelings)  -However, these negative feelings still appear fresh, almost as if re-experiencing  -Overall narrative indicates an unreadiness to address the relationship with the caregiver |
| 5 | “You know I tell you I was five years old or four turning five, washing dishes and I did it and I was getting smacked into the corner. Um no way I can’t say no way cuz I’m trying to change everything and now it’s affecting me now with my kids. You know I haven’t given myself time to heal, I had [my child] early.” | -Acknowledges past traumatic experiences and how these experiences affect her today, including her caregiving  -Expresses desire to work on her feelings associated with the relationship, but still struggles with how to address these feelings |
| 7 | “No child deserves to go through that, I don’t care. You know, um, it taught me that um you can’t treat children or anybody like, like that, like how I was treated so that helped me. Like I think it did help me because then I know that that’s not the right way to be. Otherwise um children just emulate what how their parents were and all that but I know that that wasn’t right from age of four to when I left there… I guess um the main thing would be that um when you think something is despairing there are people to help and and um a friend of mine helped me tremendously because she’s the one that um she was there for me when I had [my child] and said you know that I could do it and helped me quite a bit with him and I’m thankful to her for that” | -Acknowledges how her upbringing impacts her parenting  -Has taken action to begin the healing process by seeking support from a friend  -However, despite a growing confidence in herself and her parenting, the narrative suggests past transgressions still disrupt her relationship with her own caregiver; seems as if process of resolution is still in progress |
| 9 | “I don’t have to agree with [my mother’s] choices, I don’t have to support it, but at least listen to it and try to understand at least where the other person’s coming from. So, in that respect I think I’m different than my mom and wanna continue to be that way. She’s learned over the years though” | -Shows notable perspective taking and acceptance  -Acknowledges change in the relationship  -Shows optimism regarding her own caregiving capacities despite her negative experiences  -Accompanied by consistency and coherence across the rest of the narrative |

*Note.* Evidence of high resolution should also be accompanied by coherence and consistency across the transcript, as indicated in the coding manual. The current table is designed to provide prototypical examples of *brief* excerpts that characterize sample evidence of each score. When coding, individual excerpts should always be interpreted in the context of the full interview.
